# Diurnal rhythms of heart and respiratory rates in donkeys of different age groups during the cold‐dry and hot‐dry seasons in a tropical savannah

**DOI:** 10.14814/phy2.13855

**Published:** 2018-09-05

**Authors:** Friday O. Zakari, Joseph O. Ayo, Peter I. Rekwot, Muhammed U. Kawu, Ndazo S. Minka

**Affiliations:** ^1^ Department of Veterinary Physiology Faculty of Veterinary Medicine Ahmadu Bello University Zaria Nigeria; ^2^ National Animal Production Research Institute Shika‐Zaria Nigeria; ^3^ Division of Agricultural Colleges College of Agriculture and Animal Science Ahmadu Bello University Mando‐Kaduna Nigeria

**Keywords:** Cold‐dry season, diurnal rhythm, heart rate, hot‐dry season, respiratory rate

## Abstract

The aim of this study was to determine the effect of season on diurnal rhythms of heart (HR) and respiratory rates (RespR) in the adult, yearling, and foal donkeys during the cold‐dry and hot‐dry seasons under natural light/dark cycle. The resting HR and RespR were recorded bihourly for 24 consecutive hours from 06:00 to 06:00 h (GMT +1) in 30, clinically healthy donkeys (10 adults,10 yearlings, and 10 foals). Dry‐bulb temperature (DBT), relative humidity (RH), temperature‐humidity index (THI) and wet‐bulb globe temperature index (WBGT) inside the pen were recorded bihourly from 06:00 to 06:00 h. Values of DBT, THI, and WBGT obtained during the hot‐dry season were significantly (*P *<* *0.05) higher than corresponding values recorded during the cold‐dry season. Application of single‐cosinor procedure showed that HR and RespR exhibited daily rhythmicity in both seasons. The mesors of the HR in adult (41.51 ± 0.34 beats/min [bpm]), yearling (40.80 ± 0.43 bpm), and foal (47.55 ± 0.40 bpm) donkeys during the cold‐dry season were significantly (*P *<* *0.01) lower, compared to the corresponding values of 48.4 ± 0.40 bpm, 50.42 ± 0.52 bpm and 58.10 ± 0.50 bpm, respectively during the hot‐dry season. The mesors of RespR in adult, yearling, and foal donkeys during the hot‐dry season were higher (*P *<* *0.05), when compared to the corresponding values recorded in the cold‐dry season. The HR and RespR of foals were significantly (*P *<* *0.05) higher than those of the adult and yearling donkeys. Amplitudes of HR and RespR were higher during the hot‐dry season than the cold‐dry season. In conclusion, seasonal changes affect diurnal rhythmicity of HR and RespR of adult, yearling, and foal donkeys during the cold‐dry and hot‐dry seasons. The HR and RespR of donkeys vary with age, with higher values in the foals than the adult and yearling donkeys in both seasons.

## Introduction

Donkeys support members of the poorest communities by serving as draught animals for farm work and transportation of various materials in rural and urban areas (Pritchard et al. 2005; Yilmaz and Wilson 2013). They thrive, reproduce, and are kept for their milk and meat in arid and semiarid regions under harsh environmental conditions (Polidori et al. 2008). In the northern parts of Nigeria, they are used mainly as pack animals to transport goods (Blench et al. 2004). The Northern Guinea Savannah zone of Nigeria is characterized by three distinct seasons, namely: the cold‐dry, hot‐dry, and the rainy seasons (Dzenda et al. 2011; Ayo et al. 2014). Of the three seasons in the zone, the cold‐dry and hot‐dry seasons have been described as thermally stressful to livestock (Aluwong et al. 2017). Environmental parameters that adversely affect livestock performance and health are ambient temperature (AT), relative humidity (RH), thermal radiation, and air speed (Sevi and Caroprese 2012). In the tropical and subtropical regions of the world, animals are exposed to extreme thermal environmental conditions of high ambient temperature (AT) and high relative humidity (RH), leading to heat stress (Sejian et al. 2012). Therefore, temperature‐humidity index (THI) and wet‐bulb globe temperature index (WBGT) are used as tools in evaluating the environmental thermal load on animals (Budd 2008; El‐Tarabany et al. 2017).

Animals are affected by diurnal and seasonal fluctuations of many physical factors of their environment (Piccione et al. 2003; Chan et al. 2016; Davimes et al. 2016, 2017). Diurnal and seasonal rhythms in biochemical, metabolic, endocrine, physiological, and behavioral processes are a fundamental feature of all living organisms (Piccione et al. 2005a). Adaptation capacity of mammals to the environmental changes resides in a master circadian clock located in the suprachiasmatic nuclei (SCN) of the anterior hypothalamus that serves as a relay between the environment and the body, regulating the oscillators in peripheral tissues (Silver and Kriegsfeld 2014). Information from the environment is relayed to the hypothalamus where it is integrated, enabling the animal to efficiently respond to changes in the environment (Piccione et al. 2005b, 2008; Bertolucci et al. 2008; Golombek and Rosenstein 2010; Pevet and Challet 2011; Buhr and Takahashi 2013; Husse et al. 2015). Biological rhythms are intrinsic properties of all living things and exogenous factors such as AT have impact on the biological rhythm of HR and RespR (Mortola 2004; Refinetti 2006; Gubin et al. 2017).

Physiologic responses of the animal during exposure to environmental factors have been determined by variations in cardiorespiratory responses, often measured as fluctuations in HR and RespR (Mortola 2004; Piccione et al. 2009). The fluctuations are used as indices of meteorologic stress, whereas the absence of any change serves as an index of tolerance. The HR and RespR are important physiologic indices in determining the responses of donkeys to changes in thermal environmental conditions, and variations in the parameters are indicative of adjustments to maintain homeostasis (Homma and Masaoka 2008; Seebacher et al. 2015). Extreme environmental temperature affects daily rhythms of physiological functions, which distort the circadian clock, and invariably compromise production and welfare of the animals (Minka and Ayo 2016). Variations in HR reflect the balance between sympathetic and parasympathetic tones and are used as an indicator of stress response in animals (Piccione et al. 2009; Ohmura et al. 2012; Reyes del Paso et al. 2013). Animals under natural light/dark cycle are involved in complex relationship with the environment, which results in fluctuations in biological rhythms that are not observed in temporal studies carried out under laboratory conditions (Scheibler and Wollnik 2009; Pita et al. 2011).

In summary, the study examined the effect of cold‐dry and hot‐dry seasons on HR and RespR in donkeys with the aim of providing baseline data that are currently lacking in the tropical Guinea Savannah zone. The result also demonstrated the effect of age on HR and RespR with foal donkeys having higher values. The data may be beneficial in clinical evaluation and improvement of productivity and welfare of donkeys under different thermal environmental conditions. We hypothesized that seasons affect daily rhythms of HR and RespR of the adult, yearling, and foal donkeys during the cold‐dry and hot‐dry seasons under natural light/dark cycle. We hypothesized this due to the influence of environmental parameters (DBT, RH, THI, and WBGT) on diurnal rhythmicity of HR and RespR of the donkeys. The null hypothesis that the cold‐dry and hot‐dry seasons do not affect the diurnal rhythms of HR and RespR in adult, yearling, and foal donkeys was tested and rejected.

## Materials and Methods

### Experimental location

The experiment was conducted during the peak of the cold‐dry (15th, 22nd, and 29th January, 2017) and hot‐dry (5th, 12th, and 19th April, 2017) seasons at the donkey pen of the Equine and Camel Research Programme, National Animal Production Research Institute, Shika‐Zaria (11°12′ N; 7°33′ E), located in the Northern Guinea Savannah zone of Nigeria, and at an altitude of about 610 m above sea level. Length of photophase and scotophase during the hot‐dry season were 12 h, 19 min, and 11 h, 41 min, respectively (Sunrise at 06:19 h and sunset at 18:41 h). During the cold‐dry season, the length of the photophase and scotophase were 11 h 35 min, and 12 h 25 min, respectively (Sunrise at 06:53 h and sunset at18:07 h).

### Management of animals

Thirty, clinically healthy free‐ranging donkeys belonging to National Animal Production Research Institute, Shika‐Zaria, Nigeria were used as the experimental animals. The donkeys were divided into three groups, based on age and sex: Group I: 10 foals (five males and five females); Group II: 10 yearlings (five males and five females) and Group III: 10 adults (five males and five females). Two different sets of foals of 1–2 months’ old were used in each season. They were assigned a body condition score of six (more than moderate) out of nine. The parameter of body condition, ranging from 1 to 9, indicates a donkey's nutritional status and well‐being by accessing the amount of body fat (Pearson and Ouassat 2000).

The body weight of each donkey was determined using a weighing platform scale, Avery Weigh‐tronix Platform Scale (AWB120, Egham, Surrey, UK) and expressed in kilogrammes. The weights of the foals, yearlings, and adult donkeys were 40.67 ± 2.20, 91.53 ± 0.54 and 140 ± 0.71 kg, respectively; and their corresponding average ages were 1.50 ± 0.02 months, 1.51 ± 0.01 years, and 8.03 ± 0.06 years, respectively. All the donkeys were kept under semiintensive management system. They were housed in communal pens, partly walled with open space, which exposed the animals to meteorological factors and provided adequate ventilation. The pens measured 4 × 7 m wide and 3.5 m high from the floor which were made of concrete and covered with straw beddings. The donkeys were kept in the pen for 6 days (3 days each, for cold‐dry and hot‐dry seasons) with an interval of 1 week, during which they were released to graze on natural pastures. During confinement, the donkeys were fed with woolly finger grass (*Digitaria smutsii*) and signal grass (*Brachiaria decumbens*) and supplemented with maize and sorghum bran. In line with standard farming practices obtained in our locality, the animals had free access to water and were fed hay at 06.00, 12.00, and 18.00 h).

### Ethics

The study was approved by the Ahmadu Bello University Committee on Animal Welfare and Use. The donkeys were handled according to the guidelines for ethical conduct in the care and use of animals developed by the American Psychological Association's Committee on Animal Research and Ethics (APA 2010).

### Thermal environmental parameters

The dry‐bulb temperatures (DBT) and wet‐bulb temperatures (WBT) were recorded bihourly for 24 consecutive hours from 06:00 to 06:00 h using a wet‐ and dry‐bulb thermometer (Mason's type, Zeal, England), and the RH was obtained using Osmond's hygrometric table (Narindra Scientific Industries, Haryana, India). The heat load on the donkeys was obtained using the THI (Yousef 1985) and WBGT (Schroter et al. 1996). The THI was calculated during the experimental period by the method of Yousef (1985:

$$\text{THI}=(0.35t_{d}+0.65t_{\mathrm{wb}})\times 1.8+32$$

Where, *t*_d_ = dry‐bulb temperature (°C), *t*_wb_= wet‐bulb temperature (°C).

The WBGT was derived from DBT and RH using the WBGT index chart, obtained from the National Weather Service, Tulsa, Oklahoma, USA. The meteorological data of wind speed, wind direction, and solar radiation from the study period were collated from the Nigerian Meteorological Agency, Zaria, Nigeria, located at a distance of 2 km from the experimental site.

### Evaluation of HR and RespR

The experiment was conducted during the cold‐dry (November–February) and hot‐dry (March–April) seasons by measuring the HR and RespR (Dzenda et al. 2013, 2015). Measurements of the HR and RespR were taken at 2 h intervals (bihourly) for 24 consecutive hours from 06:00 h on day 1 and ending at 06:00 h (GMT +1) on day 2 for each experimental day. Briefly, each donkey was restrained lightly while measurements were taken. The RespR of 30 donkeys were taken by observing and counting the number of respiratory flank movements during 1 min. The researchers (two in number) and eight trained personnel commenced the observation and recordings simultaneously at 06:00 h. Each individual was responsible for recording the RespR of three donkeys. The whole procedure of RespR measurement lasted for 30 min.

The resting HR was recorded using the Polar Equine HealthCheck FT1 HR Monitor (Model 93045117, Warminster, Pennsylvania, USA) comprising a combination of a Polar FT1 training computer and a Polar Equine T31 Chest Transmitter (a sensor with a handle‐bar). The T31 transmitter sent the HR signal to the training computer and the HR was then displayed on the screen of the computer. The handle‐bar/transmitter was placed at the level of the fourth and fifth ribs on the left side of each donkey, after wetting the area with water using a sponge. The FT1 training computer was kept 1 m away from the handle‐bar. The donkey's HR was displayed 5 sec after pressing the button to start (Khelifi et al. 2017). Six individuals (three researchers and three trained personnel) commenced the measurements and recordings simultaneously. Each individual was responsible for recording the HR of five donkeys. Briefly, each donkey was restrained lightly and HR of 30 donkeys was recorded with a Polar Equine HealthCheck FT1 HR Monitor during 2.5 min. Since the donkeys were very docile, restraining of each of the 30 animals took 20 sec. The whole procedure of HR measurement lasted for 12.5 min.

### Statistical analysis

Data obtained were expressed as mean ± standard error of the mean (Mean ± SEM) and subjected to the D'Agostino‐Pearson Omnibus normality test. Data were found to be normally distributed. Cosinor analysis was used to determine the diurnal rhythms of HR and RespR of the donkeys. The mesor (rhythm‐adjusted mean), amplitude (calculated as half the maximum‐minimum range of the oscillation), acrophase (time of peak), robustness (strength of rhythmicity) computed as the fraction of the variance and the period was fixed at 24 h. Values were subjected to one‐way analysis of variance (ANOVA), followed by Tukey's multiple comparison test to compare differences in age groups. Comparison between cold‐dry and hot‐dry seasons was evaluated using Student's *t* test. GraphPad Prism 6.0 for Windows (GraphPad Software, San Diego, CA) was used to compare the difference between means in different age groups and seasonal variations. Pearson's correlation analysis was used to evaluate the relationships between thermal environmental parameters, HR, and RespR. Values of *P *<* *0.05 were considered significant.

## Results

### Characteristics of rhythmic parameters in HR during the cold‐dry and hot‐dry seasons

**Figure 1:**
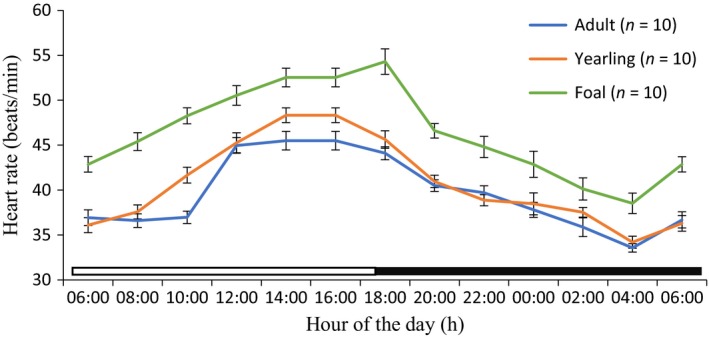
Daily rhythms of heartbeats in adult, yearling, and foal donkeys during the cold‐dry season under natural conditions. Each data point represents the mean ± SEM of 10 donkeys at each period of measurements. The horizontal bars denote the light and dark phases of the prevailing light‐dark cycle. For each season measurements were made at 2‐h intervals for a period of 24 h.

**Figure 2:**
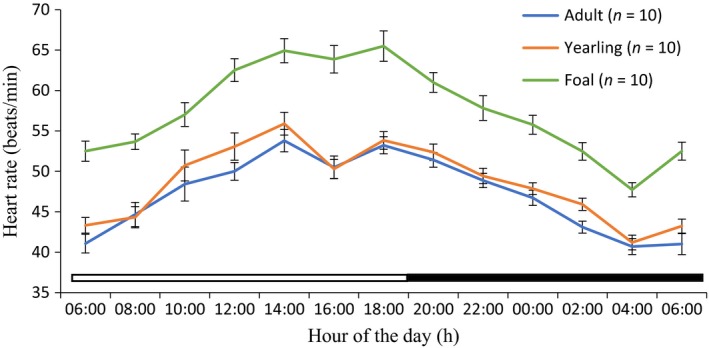
Daily rhythms of heartbeats in adult, yearling, and foal donkeys during the hot‐dry season under natural conditions. Each data point represents the mean ± SEM of 10 donkeys at each period of measurements. The horizontal bars denote the light and dark phases of the prevailing light‐dark cycle. For each season measurements were made at 2‐h intervals for a period of 24 h.

Rhythmic parameters of mesor, amplitude, acrophase, and robustness are shown in Table 1. Application of cosine model showed that the HR of the adult, yearling, and foal donkeys exhibited strong diurnal rhythms during the cold‐dry and hot‐dry seasons (Figs. 1, 2). The mesors of the HR of adult (41.51 ± 0.34 bpm), yearling (40.80 ± 0.43 bpm), and foal (47.55 ± 0.40 bpm) donkeys during the cold‐dry season were (*P *<* *0.01) lower compared to the corresponding values of 48.4 ± 0.40, 50.42 ± 0.52 and 58.10 ± 0.50 bpm during the hot‐dry season. The amplitude of diurnal rhythm of adults, yearlings, and foals were significantly (*P *<* *0.05) higher during the hot‐dry season than the cold‐dry season (Table 1). The acrophase of foal donkeys (18:10–19:40) was delayed (*P *<* *0.05) when compared to those of the yearling (16:00–15:45 h) and adult donkeys (14:20–15:45 h) in both seasons. The robustness in the adult, yearling, and foal donkeys in the hot‐dry season did not differ (*P *>* *0.05) from the corresponding values recorded during the cold‐dry season.

**Table 1:** The mesor, amplitude, acrophase, and robustness of the heart rate and respiratory rate during the cold‐dry (harmattan) and hot‐dry season

	Cold‐dry season			Hot‐dry season		
	Adult (*n* = 10)	Yearling (*n* = 10)	Foal (*n* = 10)	Adult (*n* = 10)	Yearling (*n* = 10)	Foal (*n* = 10)
Mesor						
HR (beats/min)	41.51 ± 0.34^a,1^	40.80 ± 0.43^a,1^	47.55 ± 0.40^b,1^	48.41 ± 0.40^a,2^	50.40 ± 0.52^a,2^	58.10 ± 0.50^b,2^
RR (cycles/min)	21.65 ± 0.25^a,1^	22.58 ± 0.30^a,1^	26.54 ± 0.43^b,1^	26.42 ± 0.35^a,2^	26.58 ± 0.35^a,2^	32.42 ± 0.32^b,2^
Amplitude						
HR (beats/min)	16.00 ± 0.94^a,1^	16.00 ± 1.10^a,1^	24.00 ± 1.50^b,1^	26.00 ± 2.02^a,2^	28.00 ± 1.80^a,2^	32.50 ± 2.50^b,2^
RR (cycles/min)	11.00 ± 1.10^a,1^	10.00 ± 0.78^a,1^	18.00 ± 1.50^b,1^	14.00 ± 1.40^a,2^	11.00 ± 0.90^a,2^	22.00 ± 1.15^b,2^
Acrophase						
HR (beats/min)	14:20 ± 0.10^a,1^	16:00 ± 0.15^a,1^	18:10 ± 0.14^b,1^	15:40 ± 0.11^a,2^	15:45 ± 0.12^a,1^	19:40 ± 0.12^b,2^
	(46.00 ± 4.25)	(48.50 ± 8.15)	(54.00 ± 5.10)	(54.40 ± 6.50)	(56.00 ± 5.51)	(65.50 ± 8.91)
RR (cycles/min)	14:20 ± 0.14^a,1^	14:40 ± 0.15^a,1^	14:50 ± 0.10^a,1^	14:20 ± 0.10^a,1^	14:10 ± 0.20^a,1^	14:10 ± 0.16^a,1^
	(28.00 ± 2.15)	(28.00 ± 3.14)	(32.00 ± 3.34)	(30.00 ± 4.14)	(32.20 ± 5.14)	(37.20 ± 4.00)
Robustness (%)						
HR (beats/min)	87.40 ± 19.11	92.80 ± 25.34	93.80 ± 22.55	92.40 ± 20.22	83.50 ± 20.11	95.10 ± 25.12
RR (cycles/min)	88.80 ± 16.44	87.80 ± 17.44	90.20 ± 21.33	86.30 ± 23.22	86.40 ± 24.22	93.50 ± 22.11

Values are expressed as mean ± SEM. Values with different alphabets and figures differ significantly (*P *<* *0.05). ^a, b^:between adult, yearling, and foal donkeys, ^1,2^: between seasons.

### Characteristics of rhythmic parameters RespR during the cold‐dry and hot‐dry seasons

**Figure 3:**
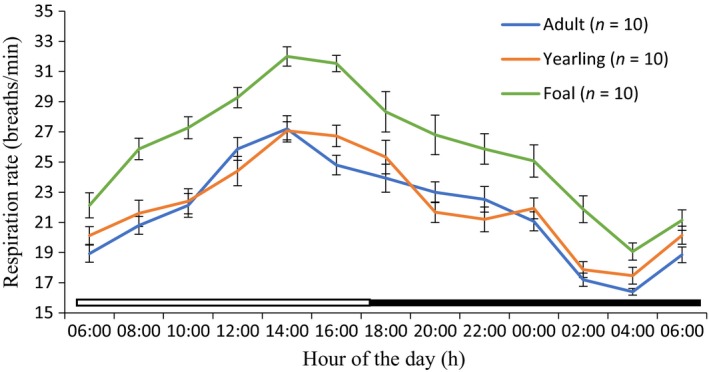
Daily rhythms of respiration rate in adult, yearling, and foal donkeys during the cold‐dry season under natural conditions. Each data point represents the mean ± SEM of 10 donkeys at each period of measurements. The horizontal bars denote the light and dark phases of the prevailing light–dark cycle. For each season measurements were made at 2‐h intervals for a period of 24 h.

**Figure 4:**
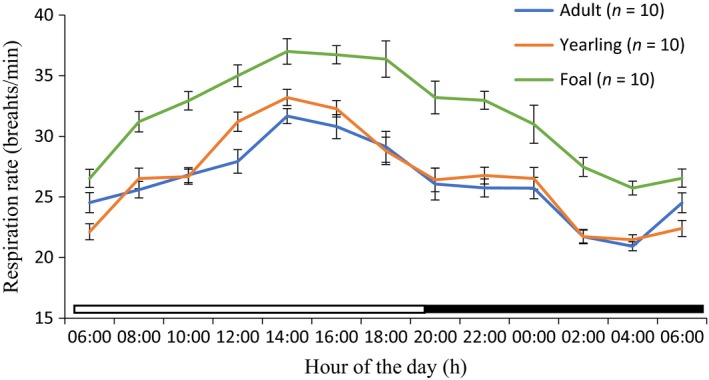
Daily rhythms of respiration rate in adult, yearling, and foal donkeys during the hot‐dry season under natural conditions. Each data point represents the mean ± SEM of 10 donkeys at each period of measurements. The horizontal bars denote the dark and light phases of the prevailing light–dark cycle. For each season measurements were made at 2‐h intervals for a period of 24 h.

Rhythmic parameters of mesor, amplitude, acrophase, and robustness are shown in Table 1. The application of cosine model showed that the RespR of the adult, yearling, and foal donkeys during the cold‐dry and hot‐dry seasons exhibited strong diurnal rhythms (Figs. 3, 4). The mesors in adult (26.42 ± 0.35 cycles per minute [cpm]), yearling (26.58 ± 0.35 cpm), and foal (32.42 ± 0.32 cpm) donkeys during the hot‐dry season were higher (*P *<* *0.05), when compared to the corresponding values of 21.65 ± 0.25, 22.58 ± 0.30 and 26.54 ± 0.43 cpm, recorded in the cold‐dry season. The amplitudes of the adult and yearling donkeys were not significantly different in both seasons. The rhythmic parameters of the acrophase in the adult, yearling, and foal donkeys were between 14.00 and 14.50 h during the photophase of the cold‐dry and hot‐dry seasons (Table 1). The robustness in the adult, yearling, and foal donkeys in the hot‐dry season did not differ (*P *>* *0.05) from the corresponding values recorded during the cold‐dry season.

### Thermal environmental parameters from the study period

**Figure 5:**
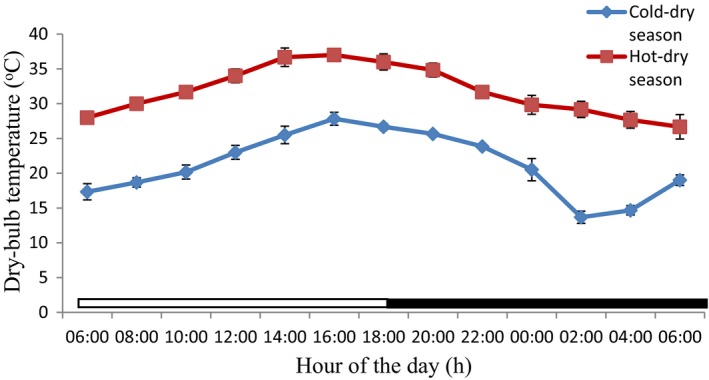
Daily rhythms in dry‐bulb temperature during the cold‐dry and hot‐dry seasons. The horizontal bars denote the light and dark phases of the prevailing light–dark cycle. For each season measurements were made at 2‐h intervals for a period of 24 h.

**Figure 6:**
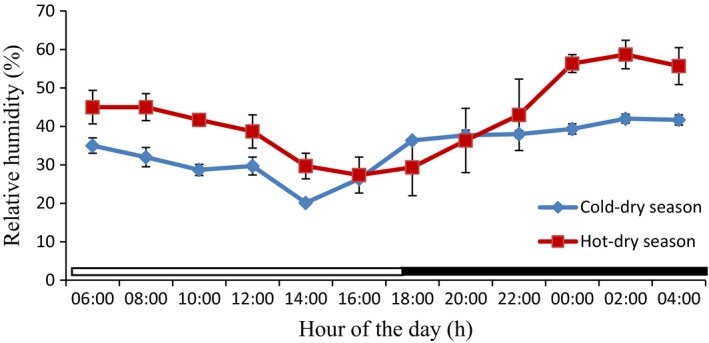
Daily rhythms in relative humidity during the cold‐dry and hot‐dry seasons. The horizontal bars denote the light and dark phases of the prevailing light‐dark cycle. For each season measurements were made at 2‐h intervals for a period of 24 h.

**Figure 7:**
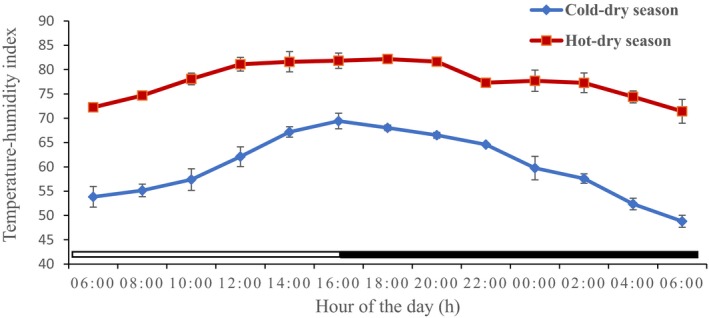
Daily rhythms in temperature‐humidity index during the cold‐dry and hot‐dry seasons. The horizontal bars denote the light and dark phases of the prevailing light‐dark cycle. For each season measurements were made at 2‐h intervals for a period of 24 h.

**Figure 8:**
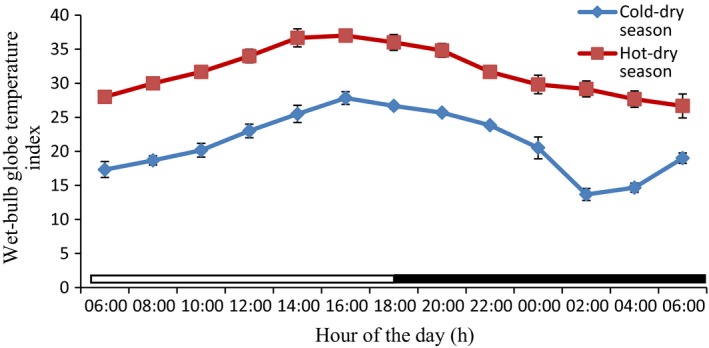
Daily rhythms in wet‐bulb globe temperature index during the cold‐dry and hot‐dry seasons. The horizontal bars denote the light and dark phases of the prevailing light–dark cycle.For each season measurements were made at 2‐h intervals for a period of 24 h.

The values of the thermal environmental parameters obtained inside the donkey's pen during the experimental period are shown in Table 2, and Figures 5, 6, 7 and 8. The mean, extreme maximum, and extreme minimum DBT values (31.78 ± 0.61°C, 38.00°C and 24.00°C, respectively), recorded during the hot‐dry season were higher (*P *<* *0.001) than the corresponding values (21.27 ± 0.74°C, 29.00°C and 12.00°C, respectively) obtained during the cold‐dry season. During the hot‐dry season, the DBT rose progressively from a relatively low value of 30.00 ± 0.00°C at sunrise (8:00 h), attained a peak value of 37.00 ± 0.29°C at 16:00 h; and thereafter fell slowly through the late evening. The mean, extreme maximum, and extreme minimum RH values of 44.90 ± 2.03%, 66.00% and 28.00%, recorded during the hot‐dry season were also higher (*P *<* *0.001) than the corresponding values of 35.28 ± 0.83%, 44.00%, and 24.00%, obtained during the cold‐dry season. A similar trend was observed in the WBGT and THI. The DBT, RH, and THI were higher during the light phase (photophase) than the dark phase (scotophase) of the light–dark cycle in both seasons, but RH was higher in the morning and scotophase in both seasons.

**Table 2:** Fluctuations in dry‐bulb temperature, dry‐bulb temperature, relative humidity, wet‐bulb globe temperature and temperature‐humidity indices

Hours	Cold‐dry season				Hot‐dry season			
	DBT (°C)	RH (%)	WBGT	THI	DBT (°C)	RH (%)	WBGT	THI
06:00	15.00–18.50	31.00–37.00	15.00–18.00	49.64–56.53	27.00–29.00	38.00–53.00	26.00–27.00	70.70–73.67
08:00	18.00–20.00	28.00–30.00	17.00–18.00	53.87–57.74	30.00–30.00	40.00–45.00	28.00–28.00	74.30–75.47
10:00	18.50–22.00	28.00–30.00	16.00–20.00	54.19–61.66	31.00–32.00	41.00–52.00	29.00–31.00	76.10–80.24
12:00	21.00–24.00	25.00–32.00	18.00–21.00	58.10–64.67	33.00–36.00	30.00–43.00	31.00–31.00	79.70–83.93
14:00	23.00–27.00	24.00–29.00	20.00–25.00	65.21–68.90	34.00–38.00	28.00–33.00	30.00–33.00	77.99–85.19
16:00	26.00–29.00	34.00–35.00	23.00–27.00	67.10–72.50	36.50–37.50	28.00–36.00	30.00–35.00	79.57–84.88
18:00	26.00‐27.50	35.00–37.00	23.00–25.00	67.10–69.00	34.00–38.00	29.00–44.00	30.00–33.00	80.99–84.02
20:00	25.00–26.00	35.00–39.00	22.00–23.00	65.30–67.10	33.00–36.50	28.00–53.00	30.00–33.00	81.00–82.04
22:00	18.00–23.50	38.00–38.00	22.00–22.00	64.36–64.67	30.00–33.00	30.00–61.00	29.00–31.00	76.19–78.98
00:00	18.00–23.50	38.00–42.00	18.00–22.00	56.21–64.36	28.00–32.50	54.00–61.00	28.00–33.00	74.21–81.73
02:00	12.00–15.00	40.00–44.00	18.00–20.00	56.21–59.45	28.00–31.50	54.00–66.00	28.00–32.00	74.21–81.10
04:00	14.00–16.00	39.00–43.00	16.00–17.00	50.18–54.28	26.00–30.00	49.00–65.00	27.00–29.00	72.41–76.64
06:00	18.00–20.50	35.00–40.00	14.00–15.00	46.58–50.81	24.00–30.00	45.00–58.00	23.00–28.00	67.01–75.47
Mean ± SEM	21.27 ± 0.74^1^ (12.00–29.00)	35.28 ± 0.83^1^ (24.00–44.00)	19.82 ± 0.54^1^ (14.00–27.00)	60.20 ± 1.09^1^ (46.58–72.50)	31.78 ± 0.61^2^ (24.00–38.00)	44.90 ± 2.03^2^ (28.00–66.00)	29.69 ± 0.38^2^ (23.00–35.00)	77.81 ± 0.68^2^ (67.01–85.19)

Values in parenthesis are minimum and maximum. Values with different superscript figures within rows are significantly (*P *<* *0.05) different. DBT; dry‐bulb temperature, RH; relative humidity, WBGT; wet‐bulb globe temperature index; THI; temperature‐humidity index.

### Relationship between thermal environment and HR and RespR

Correlation coefficients (Pearson's, *r*) between thermal environmental parameters, HR, and RespR in adult, yearling, and foal donkeys are shown in Table 3. On the overall, HR of the donkeys was positively correlated (*P *<* *0.001) with DBT, WBGT, and THI, but negatively correlated with RH (*P *<* *0.01). Similarly, RespR of donkeys was positively correlated (*P *<* *0.001) with DBT, WBGT, and THI, but negatively correlated with RH (*P *<* *0.01) (Table 3). The relationship between photoperiod (photophase and scotophase) with HR and RespR are shown in Table 3.

**Table 3:** The relationship between thermal environmental parameters and the heart and respiratory rates in donkeys

	Correlation coefficient, *r*			
	Adult (*n* = 10)	Yearling (*n* = 10)	Foal (*n* = 10)	Overall (*n* = 30)
Dry‐bulb temperature and heart rate	0.6690***	0.7470****	0.7806***	0.7333****
Wet‐bulb globe temperature index and heart rate	0.7582****	0.7620***	0.7761***	0.7582****
Relative humidity and heart rate	−0.3251**	−0.2972**	−0.2918**	−0.3140**
Temperature‐humidity index and heart rate	0.7470****	0.7530****	0.7606****	0.7805****
Dry‐bulb temperature and respiratory rate	0.5175***	0.5130**	0.5259***	0.6187***
Wet‐bulb globe temperature index and respiratory rate	0.5089***	0.5114**	0.5184***	0.6913***
Relative humidity and respiratory rate	−0.0198^ns^	−0.1107^ns^	−0.0984^ns^	−0.3981**
Temperature‐humidity index and respiratory rate	0.5073***	0.5043**	0.5102**	0.6693***
Photophase and heart rate	0.6512***	0.6352***	0.7144****	0.7993****
Scotophase and heart rate	−0.7404****	−0.6273***	−0.4802**	−0.4392**
Photophase and respiratory rate	0.7238***	0.5614**	0.6676****	0.4371***
Scotophase and respiratory rate	−0.5170	−0.4361*	−0.6514***	−0.5484***

*P <* *0.05, **P <* *0.01, ***P <* *0.001, ****P <* *0.0001, ^ns^ *P *>* *0.05.

## Discussion

### HR in adult, yearling, and foal donkeys during the cold‐dry and hot‐dry seasons

The results of this study showed variation in HR values in adult, yearling, and foal donkeys during the cold‐dry and hot‐dry seasons. The HR values in adult, yearling, and foal donkeys increased gradually during the photophase of the light/dark cycle, while a decline in HR was recorded in the donkeys during the scotophase of the light/dark cycle. The diurnal fluctuation in HR during the cold‐dry and hot‐dry seasons followed the same trend as DBT, THI, and WBGT with relatively lower values during the cold‐dry than the hot‐dry season. The RH, however, peaked during the scotophase of the light/dark cycle during the cold‐dry and hot‐dry seasons. The positive correlation observed between HR with DBT, WBGT, and THI indicated that environmental parameters highly influenced the pattern of the seasonal variation in HR of the donkeys. This finding suggests that the higher DBT and THI induced higher HR, which agrees with the findings of Ayo et al. (2014) that thermal environmental conditions induce an increase in HR values during the seasons in the Northern Guinea Savannah zone. Environmental conditions such as photoperiod and environmental temperature are also known to influence the diurnal rhythms of physiological parameters. In a strict sense, diurnal rhythms in HR of homeotherms are endogenously generated, but they can be modulated by external cues, primarily daylight (Ayo et al. 2008).

The finding in the current study also agrees with that obtained by Brinkmann et al. (2012) that the mean HR of Shetland ponies varies with season: with low and high values occurring during the winter and summer periods, respectively. The peak HR in horses and Shetland ponies in the control groups, reported by Brinkmann et al. (2012) was higher than that observed in the current study. The result of HR fluctuations demonstrated that physiological parameters are vital in the evaluation of responses by equids to variations in thermal environmental conditions (Minka and Ayo 2007; Ayo et al. 2014). The HR values in adult and yearling donkeys during the cold‐dry season were within the normal range (38–45 bpm), established for donkeys (Svendsen 2008). The extreme maximum or peak value of HR in adult, yearling and foal recorded during the cold‐dry season were above the normal range (38–45 bpm), reported in tropical and subtropical regions of the world (Fielding and Krause 1998). Similarly, the mesor of HR in adult, yearling, and foals during the hot‐dry season were above the normal range (38–45 bpm) for donkeys in the tropics (Fielding and Krause 1998). The lower HR in the adult and yearling donkeys, when compared to those of the foals may be due to the increased myocardial mass and slow metabolic rate in older donkeys. The higher HR recorded in adult, yearling, and foal donkeys during the hot‐dry season may be attributed to the increased cardiac output to dissipate the excess heat load (Ohmura and Jones 2017). The increase in HR is an attempt by the animal to increase the loss of excessive heat (McManus et al. 2014). The HR of domestic donkeys subjected to transportation in Iran (Samimi 2017) was consistent with the results of the current study.

The significant elevation in donkeys’ HR during the hot‐dry season indicates that the donkeys employed increased blood flow to the body surface as a means of losing heat; apparently, via radiation, conduction, and convection from the skin surface to the atmosphere (Lenis Sanin et al. 2016). The significantly higher mesor of HR in foals demonstrated that younger and smaller donkeys have higher metabolic rates than adult donkeys (Glazier 2009). The result showed that the amplitudes in HR in foals were higher than those of the adult and yearling donkeys during the cold‐dry and hot‐dry seasons. The high amplitude of HR in foals over adult and yearling donkeys demonstrated that fluctuations were greatest in foals. The wide range of amplitude in foals suggests that the foals are most susceptible to changes in thermal environmental conditions. The mesor of HR decreased with age in foal > yearling > adult donkeys in the current study. The result agrees with the finding of Ohmura and Jones (2017), who demonstrated that HR decreases with age in foals to old horses in thoroughbred breeds. Variations in HR, which are regulated by the autonomic nervous system, may be associated with metabolic rate in equids (Ohmura et al. 2012). In general, small and young animals have higher HR than older animals as a result of the increase in body surface area to volume ratio (Speakman 2005).

The baseline HR reported by Kang and Park (2017) in 3–9‐year‐old Jeju crossbreed mares are similar to the values recorded during the cold‐dry season but lower than those obtained during the hot‐dry season. Merkies et al. (2016) reported higher HR in weaned foals than those recorded in foal donkeys in the current study. The resting mean HR values of 2‐year‐old standardbred horses, obtained in December in Sweden (Ringmark et al. 2015) was lower than those recorded in the current study in both seasons. Similarly, the HR values (during morning and afternoon periods, respectively) in 9‐year‐old Italian saddle horse (Piccione et al. 2009) were lower than those recorded in adult, yearling, and foal donkeys. The HR values in the current study were higher than the HR baseline values of different breeds (Warmblood, Australian Stock Horses, Clydesdale‐crosses, Thoroughbred, Percheron, Andalusians and Appaloosa) of horses in Australia, reported by Fenner et al. (2016). The peak values, which were attained mostly from 14:20 h to 19:40 h during the photophase period, disagree with the finding of Piccione et al. (2009), who recorded peak values between 19:00 and 20:00 h in horses. The difference in HR may be due to time of feeding and the environmental factors under natural light/dark cycle which are not constant. Thus, entrainment by time of feeding and variations in environmental factors may be responsible for the differences in HR under tropical conditions. The HR reported by Samimi and Tajik (2017) in miniature donkey breeds in Iran was higher than values obtained in the present study. The photoperiodic time shift to shorter days, apparently, contributed to the differences in acrophase of HR observed in the adult, yearling, and foal donkeys during the cold‐dry and hot‐dry seasons. The delayed acrophase in foal donkeys may be due to the immaturity of the diurnal rhythmicity of HR.

### RespR in adult, yearling, and foal donkeys during the cold‐dry and hot‐dry seasons

The result of the present study showed that diurnal rhythm of RespR varies with season, with amplitude of the rhythm differing between the cold‐dry and hot‐dry seasons, suggesting that the diurnal rhythm of RespR was influenced by seasonal factors. The RespR of the adult, yearling, and foal donkeys fluctuated during the cold‐dry and hot‐dry seasons, with apex and nadir values recorded during the photophase and scotophase of the light/dark cycle, respectively. The diurnal fluctuation in RespR during the cold‐dry and hot‐dry seasons followed the same trend as DBT, THI, and WBGT.

The results of the present demonstrated that the hot‐dry season was more thermally stressful to the pack donkeys than the cold‐dry season, and that the donkeys have successfully adapted to the seasons. The mesor of RespR in adult, yearling, and foal donkeys were within the normal range of 20–30 cpm, established for donkeys in the tropical/subtropical regions (Fielding and Krause 1998) and 13–31 cpm in the temperate region (Svendsen 2008), although the mesor of RespR in foal donkeys during the hot‐dry season was slightly above the normal range. The relative increase in mesor of RespR in the foal donkeys above the normal range of 20–30 cpm (Fielding and Krause 1998) during the hot‐dry season may be due to respiratory evaporative heat‐loss mechanism triggered by the hypothalamus to ensure homeothermy. The finding supports the result obtained by Robertshaw (2006), suggesting that elevated RespR increases heat loss from the lungs by evaporation.

The RespR reported in mares and gelding during the winter and spring periods in Romania are lower than the corresponding values in the current study (Popescu et al. 2012). The RespR recorded by Samimi and Tajik (2017) in miniature donkeys was lower than the values obtained in the current study. The relatively high RespR during the hot‐dry season compared to the cold‐dry season was apparently the result of exposure to heat, accompanied by marked alterations in respiration to increase ventilation (Zila and Calkovska 2011; Lenis Sanin et al. 2016). The increase in mesor of RespR in the hot‐dry season is in agreement with the finding of Shawaf et al. (2018) in Shetland ponies, who reported that RespR was higher in the summer than the winter period in the eastern province of Saudi Arabia. The RespR reported by Wanderley et al. (2010) in Mangalarga‐Machador horses are similar to those of adult donkeys during the cold‐dry season in the present study.

The low DBT and RH observed during the cold‐dry season resulted in a decreased RespR which may reduce respiration (Riihimäki et al. 2008) during the cold‐dry season. The relatively low RH may result in greater loss of moisture from the skin's surface, since homeothermic animals tend to lose more body water through the skin in low RH condition (Ray et al. 2006). The RespR was apparently regulated under the cold‐dry conditions in order to reduce respiratory moisture loss and reduce the dryness of the respiratory tract (Furtado et al. 2008). The decrease in RespR of adult, yearling, and foal donkeys during the cold‐dry season may be an adaptive response to decrease further loss of body heat and water to the cold‐dry atmosphere of the cold‐dry season.

The increase in mesor of RespR of the donkeys recorded during the hot‐dry season may be due to the rise in DBT during the hot‐dry season. The increase in RespR observed may be considered the main mechanism for control of homeostasis during the hot‐dry season. Furtado et al. (2008) reported that a seasonal increase in RespR may be a physiological mechanism of heat loss in hot conditions. The increased DBT during the hot‐dry season may narrow the temperature gradient between the donkeys and the environment, decreasing sensible heat loss, and increasing pulmonary evaporative heat loss with the consequent rise in RespR. This result agrees with the finding of Pritchard et al. (2006) that heat stress is usually associated with a marked increase in RespR in donkeys and horses because respiratory water loss is enhanced by increasing the RespR.

## Conclusion

Overall, the results show that the amplitude of HR and RespR in this study fluctuated with season, suggesting that the diurnal rhythm was influenced by seasonal factors. Seasonal variation exerted significant effects on the daily rhythmicity in HR and RespR in adult, yearling, and foal donkeys under natural conditions of light/dark cycles in the Northern Guinea Savannah zone of Nigeria. The results also demonstrated the effect of age on HR and RespR with foal donkeys having higher values. The HR and RespR values may be a valuable addition to baseline data for donkeys and may be useful in the evaluation of their health status, treatment, and diagnosis of diseases during the cold‐dry and hot‐dry seasons. The null hypothesis that the cold‐dry and hot‐dry seasons do not affect the diurnal rhythms of HR and RespR in adult, yearling, and foal donkeys was tested and rejected.

## Conflict of Interest

The authors report no conflicts of interest. They alone are responsible for the content and writing of the paper.
